# Plant Methods reviewer acknowledgement 2014

**DOI:** 10.1186/s13007-015-0086-2

**Published:** 2015-09-30

**Authors:** Brian G. Forde, Michael R. Roberts

**Affiliations:** Centre for Sustainable Agriculture, Lancaster Environment Centre, Lancaster University, Lancaster, LA1 4YQ UK

## Contributing reviewers

The *Plant Methods* editorial team would like to thank all of our reviewers for their assistance with peer review in 2014.

**Doug Allen**

United States of America

**Anna Amtmann**

United Kingdom

**Stig U. Andersen**

Denmark

**Paula Andrade**

Portugal

**John Andralojc**

United Kingdom

**Ryan Austin**

Canada

**Agim Ballvora**

Germany

**Frantisek Baluska**

Germany

**Philippa Barrell**

New Zealand

**Susanne Barth**

Ireland

**Jeffrey Bennetzen**

United States of America

**Kenneth Wayne Berendzen**

Germany

**Bettina Berger**

Australia

**Christine Anne Beveridge**

Australia

**Neil Boonham**

United Kingdom

**Ljudmilla Borisjuk**

Germany

**Tristan Boureau**

France

**Othmar Buchner**

Austria

**Alexander Bucksch**

United States of America

**Ángeles Calatayud Chover**

Spain

**Sebastien Carpentier**

Belgium

**Sofia Carvalho**

United States of America

**Enrique Castro Camus**

Mexico

**Divya Chandran**

India

**Randy Clark**

United States of America

**Daniel Cosgrove**

United States of America

**Jonathan Dandois**

United States of America

**Anthony Dell**

United States of America

**Savithramma Dinesh-Kumar**

United States of America

**Michael Dingkuhn**

United States of America

**Ian Dodd**

United Kingdom

**Lloyd Donaldson**

New Zealand

**John Doonan**

United Kingdom

**Wolfgang Dröge-Laser**

Germany

**Rivka Elbaum**

Israel

**David Ellsworth**

Australia

**Jose Estevez**

Argentina

**Denis Faure**

France

**Richard Ferrieri**

United States of America

**John Finer**

United States of America

**Andrew French**

United Kingdom

**Julia Frugoli**

United States of America

**Stephen C. Fry**

United Kingdom

**David Galbraith**

United States of America

**Juan Galindo**

Spain

**Jeroni Galmés**

Spain

**Katharina Gaus**

Australia

**Marina Gavilanes-Ruíz**

Mexico

**Alan Gay**

United Kingdom

**Stanton Gelvin**

United States of America

**Ralf Gente**

Germany

**Simon Gilroy**

United States of America

**Robert Godfree**

Australia

**Dominik Grimm**

Germany

**Anthony Hall**

United Kingdom

**David Hannapel**

United States of America

**Jeremy Harbinson**

Netherlands

**Philip Harris**

New Zealand

**Toshihiro Hasegawa**

Japan

**Robert Henry**

Australia

**James Higgins**

United Kingdom

**Geoff Holroyd**

United Kingdom

**Liu Hongbo**

China

**Gregor Huber**

Germany

**Mauricio Hunsche**

Germany

**Yoshihiro Izumi**

Japan

**Andrzej Jerzmanowski**

Poland

**Giles Johnson**

United Kingdom

**Neil Jones**

United Kingdom

**Kathryn Jones**

United States of America

**Nolan Kane**

United States of America

**Fumiaki Katagiri**

United States of America

**Naohiro Kato**

United States of America

**Julia Kehr**

Germany

**Edward Kendall**

Canada

**Anna Kicherer**

Germany

**Norbert Kirchgessner**

Switzerland

**Christian Klukas**

Germany

**Karin Koehl**

Germany

**Paula Kover**

United Kingdom

**Vivek Krishnakumar**

United States of America

**Tetsuya Kurata**

Japan

**Smita Kurup**

United Kingdom

**Erh-Min Lai**

Taiwan

**Blanca Landa del Castillo**

Spain

**Tony Larson**

United Kingdom

**Angela Lausch**

Germany

**Julie Law**

United States of America

**Miriam Laxa**

Germany

**Gongke Li**

China

**De-Zhu Li**

China

**Yi Li**

United Kingdom

**Zhengbin Liu**

China

**Sanzhen Liu**

United States of America

**Guillaume Lobet**

Belgium

**Connie Lovejoy**

Canada

**Kristiina Mäkinen**

Finland

**Pal Maliga**

United States of America

**Jan Malinsky**

Czech Republic

**Jaideep Mathur**

Canada

**Gareth McKinley**

United States of America

**Marion Menzel**

Germany

**Andrew Millar**

United Kingdom

**Tony Miller**

United Kingdom

**Daniel Mittleman**

United States of America

**Daniel Molitor**

Luxembourg

**Rebecca Mosher**

United States of America

**Sergi Munné-Bosch**

Spain

**Rafael F. Muñoz-Huerta**

Mexico

**Ladislav Nedbal**

Germany

**Vladimir Nekrasov**

United Kingdom

**Benjamin Neuhäuser**

Germany

**Anthony Nguy-Robertson**

United States of America

**Jeroen Nieuwland**

United Kingdom

**Kenji Omasa**

Japan

**Dylan Owen**

United Kingdom

**Anthony Paproki**

Australia

**Christian Parisod**

Switzerland

**Boris Parizot**

Belgium

**John Passioura**

Australia

**Zhaohua Peng**

United States of America

**Christophe Périn**

France

**Roland Pieruschka**

Germany

**Radha Prasanna**

India

**Tony Pridmore**

United Kingdom

**Holger Puchta**

Germany

**Muhammad Ishaq Asif Rehmani**

Pakistan

**Patrick Rich**

United States of America

**Niki Robertson**

United States of America

**Fran Robson**

United Kingdom

**Maurizio Rossetto**

Australia

**Pierre Roumet**

France

**David Rousseau**

France

**Hitoshi Sakakibara**

Japan

**Christophe Salon**

France

**Norbert Sauer**

Germany

**Korbinian Schneeberger**

Germany

**Alexander Schulz**

Denmark

**Hamid Reza Shahbazkia**

Portugal

**John Shanklin**

United States of America

**Xavier Sirault**

Australia

**Nils Stein**

Germany

**Qi Sun**

United States of America

**Takanari Tanabata**

Japan

**Keitaro Tanoi**

Japan

**Jianmin Tao**

China

**Dana Tarkowska**

Czech Republic

**Petr Tarkowski**

Czech Republic

**Lee Tarpley**

United States of America

**Ryohei Terauchi**

Japan

**Mikko Tikkanen**

Finland

**Matthew Tucker**

Australia

**Colin Turnbull**

United Kingdom

**Alex Twyford**

United States of America

**Dolors Villegas Tort**

Spain

**Dagmar Voigt**

Germany

**Jack Vossen**

Netherlands

**Elsbeth Walker**

United States of America

**John Ward**

United States of America

**Randall Weselake**

Canada

**James Westwood**

United States of America

**Alex Whan**

Australia

**Jeffrey White**

United States of America

**Keith Williams**

United States of America

**Michael Wisniewski**

United States of America

**Zhanguo Xin**

United States of America

**John Yarbrough**

United States of America

**Ze-Chun Yuan**

Canada

**Wei Zeng**

Australia

**Wenzheng Zhang**

United States of America

**Joerg Ziegler**

Germany

